# Pedicled Anterolateral Thigh Flap for Vaginal and Perineal Reconstruction

**Published:** 2013-01-21

**Authors:** Sachin M. Shridharani, Howard D. Wang, Justin M. Sacks

**Affiliations:** Department of Plastic and Reconstructive Surgery, The Johns Hopkins University School of Medicine, Baltimore, Md

**Figure:**
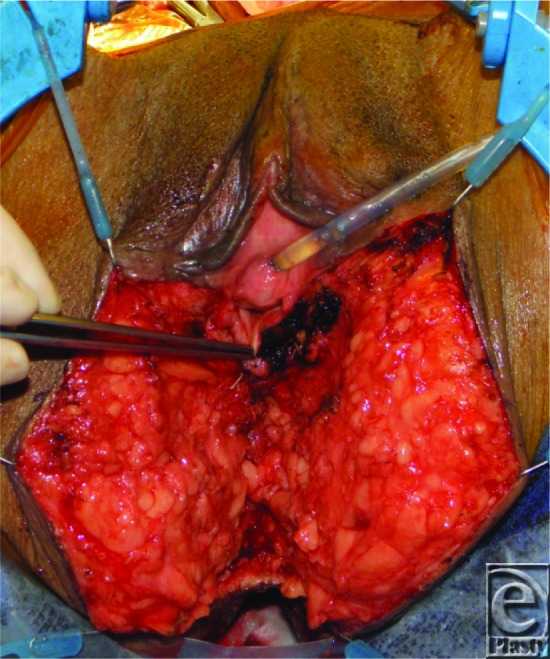


## DESCRIPTION

A 50-year-old woman with squamous cell carcinoma of the vagina and rectum underwent abdominoperineal resection and posterior vaginal wall resection. The picture appearing earlier represents the resultant defect of the tumor extirpation.

## QUESTIONS

1. **What is the classification of the ALT flap when harvested as a fasciocutaneous flap?**
2. **What is the classification of the ALT flap when harvested as a myocutaneous flap with the vastus lateralis muscle?**
3. **What is the dominant blood supply to the ALT flap?**
4. **How much pedicle length is available for rotation of the pedicled ALT flap and which areas can be reached?**
5. **Where are the cutaneous perforators most commonly located?**
6. **After elevating the pedicled ALT flap, what are the key anatomical landmarks during its transposition to the perineum?**

## DISCUSSION

This patient had a large defect with exposure of the perineum and loss of posterior vaginal wall. To reconstruct this defect, the pedicled anterolateral thigh (ALT) flap was selected. The ALT flap is a versatile flap that can be utilized in its pedicled form or as a free tissue transfer. The surgeon can choose to harvest it as either a fasciocutaneous or a myocutaneous flap. When harvested as a fasciocutaneous flap, it is generally considered a type B or type C according the Mathes-Nahai classification for fasciocutaneous flaps. It falls under these categories because of the perforators being either septocutaneous or musculocutaneous.^1^ Under the Cormack-Lamberty classification system, the ALT flap is considered a type C fasciocutaneous flap, because it has segmental perforators.^2^ When it is harvested with the vastus lateralis muscle as a myocutaneous flap, its single dominant vascular pedicle makes it a type I Mathes-Nahai myocutaneous flap.^3^

The dominant blood supply to the anterolateral skin and vastus lateralis muscle is the descending branch of the lateral circumflex femoral artery. The mean arterial and venous calibers of the pedicle are 2.1 mm and 2.3 mm, respectively. The pedicle length is generally reported as 12 cm; however, this number may vary depending on the flap design and perforator selection.^4^ The branches of the lateral femoral cutaneous nerve provide the sensory innervations to the skin of the anterolateral thigh and should be identified and preserved during dissection of the flap. In its pedicled form, the ALT flap is a versatile flap that may be employed to reconstruct the inferior posterior trunk, inferior abdomen, ipsilateral groin, ischium, anterior thigh, posterior thigh, and perineum.

**Figure 1:**
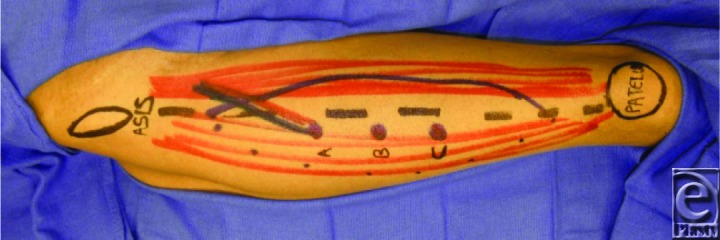
Skin markings of the ALT flap with three perforators labeled as a, b and c.

When the flap is used for perineal reconstruction, it is raised with the patient in the lithotomy position. However, surface markings should be performed with the patient in the supine position with the hip joint in a neutral position and the knee extended. In our patient, a straight line was drawn from the anterior superior iliac spine to the most lateral superior point on the patella. This line represents the cleavage plane between the rectus femoris and vastus lateralis muscle. Perforator B is located 1.5 cm lateral to the half-way point of this line (Fig 1). Peforator A and C are located 5 cm proximal and distal to perforator B, respectively.^5^ These perforator locations were confirmed with a transcutaneous Doppler. The planned skin paddle was marked in the shape of an ellipse using a template of the wound. The ellipse should be centered as distally as possible over a reliable perforator as confirmed by Doppler to reach the defect.

**Figure 2:**
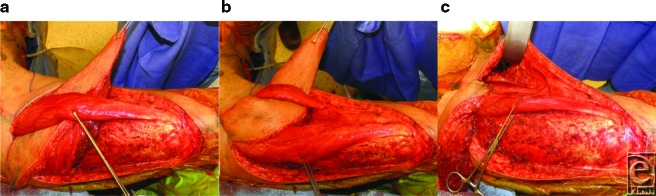
(a) Tunneling of the flap below the rectus femoris muscle. (b) Tunneling of the flap below the sartorius muscle. (c) Subcutaneous tunnel along the medial thigh for transposition of flap to perineum.

**Figure 3:**
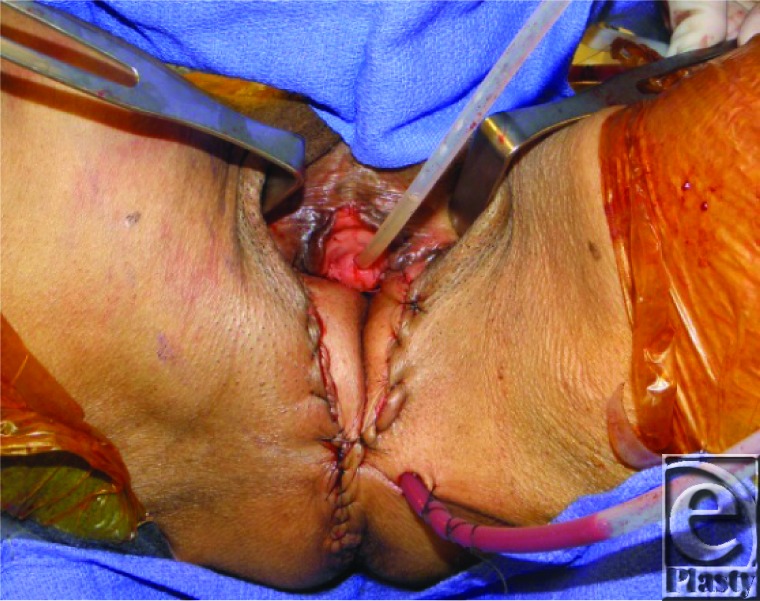
Postoperative image of the reconstructed perineum.

To assist with the obliteration of dead-space in the perineum, a cuff of vastus lateralis muscle is routinely included with the pedicled ALT. By maintaining the perforating arteries, the muscle will be sustained on the same pedicle as the ALT flap. Donor-site morbidity has been shown to be minimal when harvesting part of or all of the vastus lateralis muscle.^6^ Once the flap is islandized and elevated to the pedicle origin, the surgeon may inset the flap in nearly any direction by taking advantage of a large arc of rotation. For perineal reconstruction in our case, the ALT flap was tunneled below both the rectus femoris muscle (Fig 2a) and the sartorius muscle (Fig 2b). During this maneuver, the surgeon must take care to preserve the dominant blood supply to the rectus femoris muscle. The sartorius muscle has a Mathes-Nahai Type IV blood supply, thus segmental perforators may be ligated to create an adequate submuscular tunnel for the flap. A subcutaneous tunnel along the medial thigh was then created to gain access to the perineum for reconstruction of this area (Fig 2c). When the flap is transposed onto the perineum, it is critical that the flap's pedicle is not on any tension. The immediate postoperative image of the reconstructed perineum of our patient is shown below (Fig 3). The donor site was then primarily closed, and a small split-thickness skin graft was applied to the area of the thigh that would not close in a tension-free manner.

Using a submuscular window under the rectus femoris and sartorius and a wide subcutaneous medial thigh window, this robust flap can be successfully transferred to the perineum. By harvesting the vastus lateralis muscle along with the skin paddle, the pedicled ALT myocutaneous flap is an important tool in reconstruction of composite defects of the perineum including the posterior and lateral walls of the vagina.

## References

[1] Mathes SJ, Nahai F (1997). Reconstructive Surgery: Principles, Anatomy, and Technique.

[2] Cormack GC, Lamberty BG (1984). A classification of fascio-cutaneous flaps according to their patterns of vascularisation. Br J Plast Surg.

[3] Mathes SJ, Nahai F (1981). Classification of the vascular anatomy of muscles: experimental and clinical correlation. Plast Reconstr Surg.

[4] Wei FC, Jain V, Celik N, Chen HC, Chuang DC, Lin CH (2002). Have we found an ideal soft-tissue flap? An experience with 672 anterolateral thigh flaps. Plast Reconstr Surg.

[5] Yu P, Youssef A (2006). Efficacy of the handheld Doppler in preoperative identification of the cutaneous perforators in the anterolateral thigh flap. Plast Reconstr Surg.

[6] Hanasono MM, Skoracki RJ, Yu P (2010). A prospective study of donor-site morbidity after anterolateral thigh fasciocutaneous and myocutaneous free flap harvest in 220 patients. Plast Reconstr Surg.

